# An Enhanced Event-Based Model for Integrated Flight Safety of Fixed-Wing UAVs

**DOI:** 10.3390/s26072058

**Published:** 2026-03-25

**Authors:** Xin Ma, Xikang Lu, Hongwei Li, Xiyue Lu, Jiahua Li, Jiajun Zhao

**Affiliations:** 1 College of Air Traffic Management, Civil Aviation Flight University of China, Deyang 618307, China; maxin@cafuc.edu.cn (X.M.); xklu@cafuc.edu.cn (X.L.); 2 School of Mathematics and Statistics, Chongqing Technology and Business University, Chongqing 400067, China; lxzheng@cafuc.edu.cn; 3 AVIC Chengdu Aircraft Design & Research Institute, Chengdu 610091, China; empiresky123@sina.com

**Keywords:** mixed airspace traffic flow, precision and quantifiable safety intervals, multi-dimensional geometric features, integration of flight track planning

## Abstract

**Highlights:**

**What are the main findings?**

- An enhanced Event-based collision risk model (EMGF-M) is developed, integrating multidimensional geometric features and flight performance parameters for fixed-wing UAVs.
- The QMC-S computational method significantly improves model accuracy and stability, reducing mean error by 97.91% compared to traditional Monte Carlo methods.
- Refined three-dimensional safety separation standards are established in alignment with ICAO target safety levels, with longitudinal, lateral, and vertical intervals reduced to 4329 m, 4026 m, and 3450 m, respectively.

**What is the implication of the main finding?**

- The proposed model provides a quantifiable, model-driven basis for risk evaluation in mixed airspace operations involving manned aircraft and UAVs.
- Application in regional airport airspace design increases simulated traffic flow from 9.2 to 20.9 operations per hour, demonstrating significant improvement in airspace utilization.
- This work bridges critical gaps in current operational standards by enabling precise safety risk assessment and conflict resolution in low-altitude mixed airspace environments.

**Abstract:**

To address the issues of safety risk analysis and conflict assessment for integrated flight of manned aircraft and fixed-wing unmanned aerial vehicles (UAVs) in low-altitude mixed-operation airspace, this study enhances the foundational Event model. By incorporating UAV characteristics such as geometric features and aerodynamic mechanisms, alongside design dimensions and onboard performance metrics, an improved collision risk model is developed—the Enhanced Event-Based Framework for Multidimensional Geometry and Quasi-Monte Carlo Analysis of Flight Performance (EMGF-M). This enhancement rectifies the limitations of the basic model regarding parameter coverage and scenario adaptability, thereby improving the reliability and validity of the computational results. Experimental results demonstrate that, in accordance with the target safety level for airspace conflicts set by the International Civil Aviation Organization (ICAO), the application of the improved Event collision model yields quantifiable assessments of safety risks and safe separation distances for integrated operations in low-altitude mixed-use airspace. Utilizing these computational results for integrated flight procedure design at a general airport in Southwest China, the study shows that the air traffic flow in the low-altitude mixed-operation airspace increased from 9.2 to 20.9 operations per hour. The practical significance of this method lies in its guidance for accurately assessing safety risks in mixed airspace operations and for determining quantifiable separation minima for integrated flight trajectory planning.

## 1. Introduction

With the issuance of policy and guidance documents such as “Implementation Plan and Measures for Accelerating the Cultivation and Development of Low-Altitude Economy (2024–2027) and Several Measures” and “Interim Regulations on Flight Management of Unmanned Aircraft (State Council Order No. 761)”, reforms for low-altitude airspace are entering a period of rapid advancement. Concurrently, the formulation and validation of operational standards, including low-altitude flight rules, will also be accelerated. Currently, the main operational mode of unmanned aircraft is trial operations and airworthiness verification flights within segregated airspace for single or specific aircraft types [1]. However, due to the lack of reliable safety risk analysis and evaluation technologies, there are no standards or methods for flight path planning in mixed airspace integrated flights, which has led to gaps in safety risks, safety separations, and airspace planning methods between unmanned aircraft and manned aircraft or among multiple aircraft types, making it difficult to implement safe, scientific, and normalized integrated flights, thereby preventing the efficient development and regulation of low-altitude flights [2].

Research has found that internal and international studies on low-altitude flight safety risks and safety separations can be roughly divided into three categories. The first is basic safety risk research. Professor Reich [3] first proposed the Reich model in the field of civil aviation, deriving a basic mathematical description of the risk level of parallel flight paths. Brooker [4] proposed the Event model based on the Reich model, emphasizing its capability in safety risk analysis and handling under complex scenarios. Bai et al. [5] introduced an unmanned aircraft collision avoidance mechanism to improve the Markov model, while Zhang et al. [6] created a collision risk model for single-type unmanned aircraft in non-segregated airspace. Thus, safety risk assessment for specific aircraft types within defined airspace environments establishes the foundation for studying collision avoidance mechanisms and impact level analysis. The second is integrated safety risk assessment involving air-ground factors and multi-type aircraft operations. Deng Li et al. [7] established a basic model for the collision probability between manned and unmanned aircraft, which holds substantial implications for guiding operational safety in airport terminal areas and adjacent airspace. Wang Li Li et al. [8,9] improved collision risk by incorporating multi-source directional collision risks and random velocity distributions. Gao Yang et al. [10] developed a collision risk prediction model for general aviation and scheduled passenger aircraft under five-dimensional factors (airspace, human, aircraft, environment, and management) in airport terminal areas. Gao Jun Jie et al. [11] modified the integrated collision probability and conflict rate assessment model established by Li et al. [12], involving factors such as UAV performance characteristics, airspace conditions, operational modes, and air traffic controller elements. Consequently, the contributing factors to UAV safety risk assessment have established the foundation for multi-factor comprehensive study [13]. The third is safety separation research for specific types of unmanned aircraft based on risk. Li Hang et al. [14] focused on urban logistics unmanned aircraft collision risks, identifying key risk factors and constructing a Bayesian network model for risk assessment. Zhang Honghai et al. [15] developed an airborne collision probability model based on the characteristics of multi-rotor light UAVs, enabling the calculation of safety separations for multi-rotor unmanned aircraft in free airspace. Wang Xinglong et al. [16] discussed safety separations for eVTOL aircraft in urban low-altitude scenarios and established multiple types of safety separations. Thus, risk assessment for low-altitude UAV operations has established the foundation for focused investigation into airspace conditions and operational modes [17].

Despite the contributions summarized in Table 1, critical limitations remain. First, most improved Reich-based models still rely on simplified geometric assumptions (e.g., cuboid or spherical collision boxes) that fail to capture UAV aerodynamic profiles, leading to biased risk estimations. Second, existing comprehensive assessment models incorporate multiple factors, but factor weighting often depends on subjective expert judgment rather than empirical validation. Third, current research on specific UAV types is highly scenario-dependent, limiting its generalizability to mixed airspace operations involving both manned aircraft and diverse UAVs. Furthermore, few studies systematically integrate three-dimensional geometric features with flight performance parameters or align their results with the ICAO target level of safety (TLS).

In summary, internal and international scholars have achieved certain results in understanding the mechanisms of low-altitude flight safety risks and evaluating low-altitude flight safety separations, which provide strong guidance for constructing mathematical expressions of safety risks and summarizing collision risk factors. However, with the introduction of new policies and updates in operational needs, low-altitude flight scenarios have gradually shifted from non-segregated airspace and specific airspace to integrated flights in mixed airspace. Urban low-altitude flight mission objectives have also evolved from testing and experiments of single aircraft to multi-aircraft-type, regular operations. There is an urgent need to create more precise and quantifiable flight conflict models integrated with complete mathematical descriptions aligned with the safety target levels of the International Civil Aviation Organization (ICAO). Therefore, research on safety risks in mixed airspace integrated flights based on safety target levels, as well as safety separations and application studies oriented toward flight performance, holds significant importance. This thesis, based on the basic Event model, establishes an improved model oriented toward spatial dimensional geometric characteristics and flight performance. Through safety risk analysis of mixed airspace integrated flights, it obtains refined and quantifiable three-dimensional safety separations in space. Simulation experiments are conducted based on the operational conditions of small- and medium-sized airports in southwestern China, aiming to enhance airspace capacity through the formulation of integrated flight trajectory planning schemes [18].

## 2. Establishment of the Collision Model

### 2.1. Reich Model

**Figure 1:**
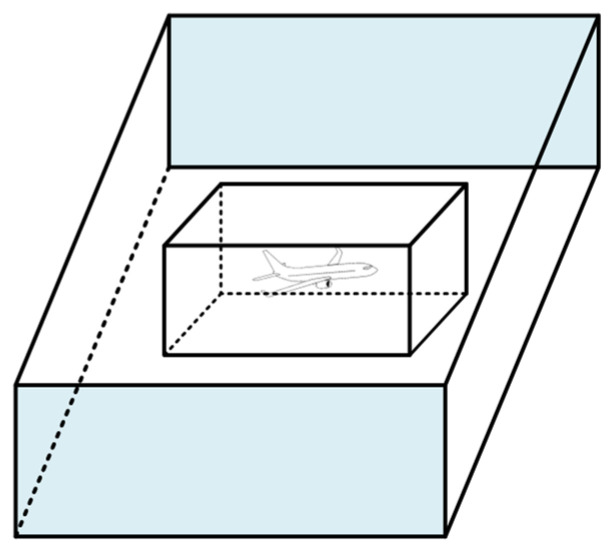
Reich base model.

The Reich model, proposed by P.G. Reich in 1966, is a foundational collision risk model for air traffic. As illustrated in Figure 1, it constructs a rectangular collision template around one aircraft and treats the other aircraft as a point mass. By calculating the probability of the point mass entering the collision template in lateral, longitudinal, and vertical dimensions, the model derives the expected number of collisions per flight hour. The detailed mathematical formulation can be found in [3].

### 2.2. Event-Based Model

**Figure 2:**
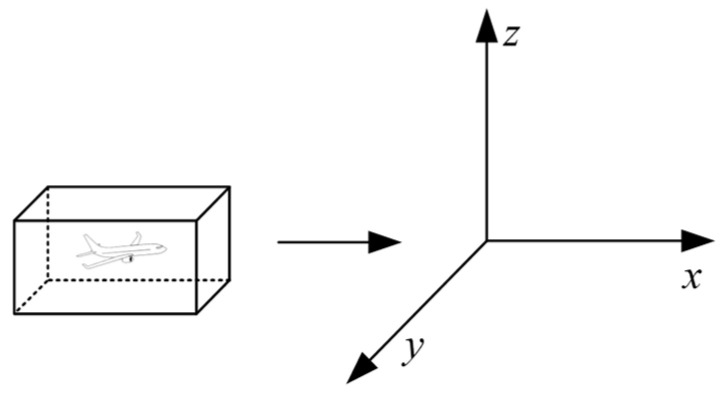
Event-based model.

**Figure 3:**
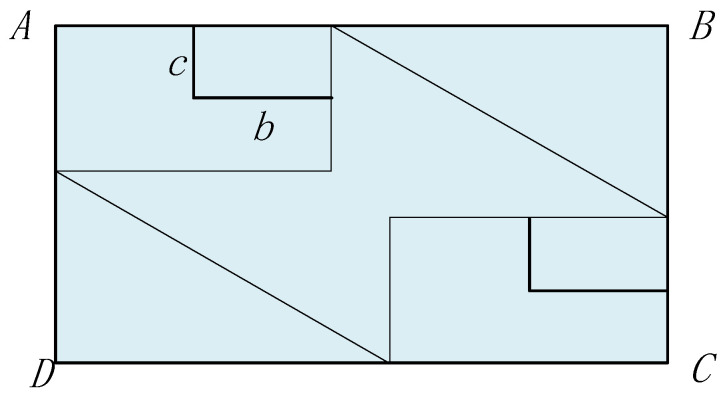
Expanded Collision Box.

Building upon the Reich model, Brooker [4] proposed the Event model to improve parameter preprocessing and incorporate aircraft motion characteristics. As shown in Figure 2 and Figure 3, the model introduces an expanded collision box when an aircraft traverses the interval layer, enabling a more accurate calculation of collision risk. The core longitudinal collision risk expression is given as follows:

Longitudinal collision model:

(1) $$N_{x}=2{f_{GERH}}_{x}\times E(0)P_{z}(0)\frac{b}{S_{y}}(1+\frac{2au_{y}}{2bu_{x}})(1+\frac{2au_{z}}{2cu_{x}})\times Q$$

where $f_{{GERH}_{x}}$ is the frequency of losing lateral separation, $P_{z}\left(0\right)$ is the lateral overlap probability, $u_{x},u_{y},u_{z}$ are relative velocities, and *a*, *b*, *c* are aircraft dimensions.

### 2.3. Establish an Improved Event Model

According to the analysis of the Reich and Event basic models, both models can calculate collision frequencies. At the same time, due to the design of the extension box, the frequency calculation results include flight status factors, which improves the reliability of the calculation results. However, due to the geometric characteristics of the conflict template, there is still room for refinement. This is reflected in the optimization and improvement of the mathematical description of aerodynamic characteristics and airborne navigation accuracy during aircraft flight, further improving the important influencing factors of collisions and their weights.

**Figure 4:**
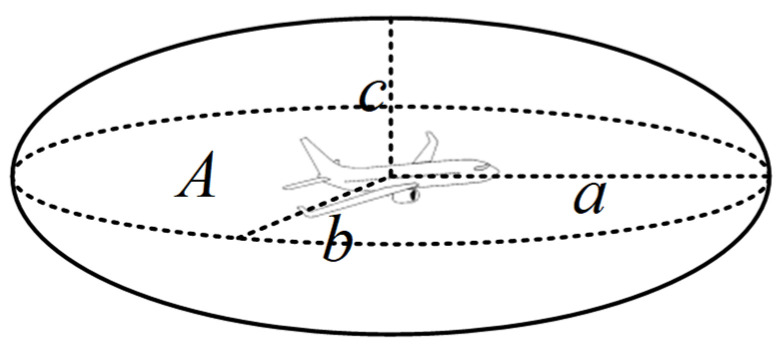
Ellipsoid collision box.

In the creation of collision boxes for the Event model, traditional collision boxes include cuboids and spheres. Cuboid collision boxes require handling of corners during calculations, making it difficult to accurately fit objects or data distributions with continuous curved boundaries, which can lead to unstable calculation results. Spherical collision boxes have excessive vertical dimensions, which do not align with the physical characteristics of aircraft. Ellipsoidal collision boxes, however, have three distinct semi-axis parameters, offering high flexibility and the feasibility of constructing a complete mathematical description [19]. They can more precisely describe the spacing between aircraft, thereby reducing errors and improving airspace utilization. Therefore, this study adopts an ellipsoidal collision box to better capture aircraft geometric characteristics. The length, width, and height of aircraft A are denoted as a, b, and c, respectively. The collision box is established using twice the dimensions of aircraft A, as shown in Figure 4:

Improved Event Model for Multidimensional Geometry and Flight Performance-MGFPE: Using the center of gravity of the passenger aircraft as the center of mass, and the ellipsoid parameters *a*, *b*, *c* as the collision box, in a pair of colliding aircraft, aircraft A is regarded as collision box A, and aircraft B is treated as a point mass on the separation layer. Based on the flight state and performance of A, displacement changes along the x, y, and z directions are established. When crossing the separation layer, if B is on A’s crossing path, it is considered a multidimensional collision. Let the longitudinal, lateral, and vertical component velocities of the two aircraft be u_x_, u_y_, and u_z_, respectively. When the collision box enters the separation layer region and crosses the separation layer, an extended collision box is generated. However, traditional Event models face challenges such as high computational complexity and low accuracy when calculating Q, leading to significant computational errors. We innovatively propose a hybrid computational model QMC-S, based on Quasi-Monte Carlo (QMC) and Scrambling methods, combined with the MGFPE model to establish the EMGF-M model. By using Halton low-differential sequences to replace traditional random numbers, we reduce clustering phenomena among samples. The introduction of perturbation methods reduces the error in computational results, making the results more stable. The improved QMC-S method is applied to calculating multi-dimensional collision risks, where the collision risk is defined as the product of the frequency of Aircraft A crossing the separation layer and the probability of Aircraft B appearing in the extended collision box.

Halton sequence calculation formula:

(2) $$x_{a}=(\Phi_{p_{1}}(a),\Phi_{p_{2}}(a),\Phi_{p_{3}}(a)\ldots \Phi_{p_{n}}(a))$$

By establishing the EMGF-M, random permutations are performed on the binary representation of low-differential sequences to break the strict deterministic structure of the sequences. Compared with traditional Monte Carlo methods, the QMC-S model can improve the flexibility and robustness of low-differential sequences. The following table shows the calculation results when comparing the QMC-S model with the traditional MC model:

As shown in the table above, compared with MC, QMC-S reduced the average error by 97.91% and the standard deviation of error by 97.57%. The calculation results show that the QMC-S model significantly reduces the average error and standard deviation of error, thereby enhancing the stability of the calculation results.

**Figure 5:**
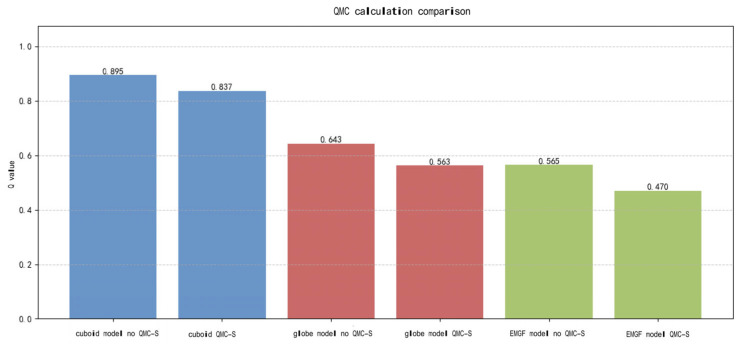
QMC-S computational comparison.

**Figure 6:**
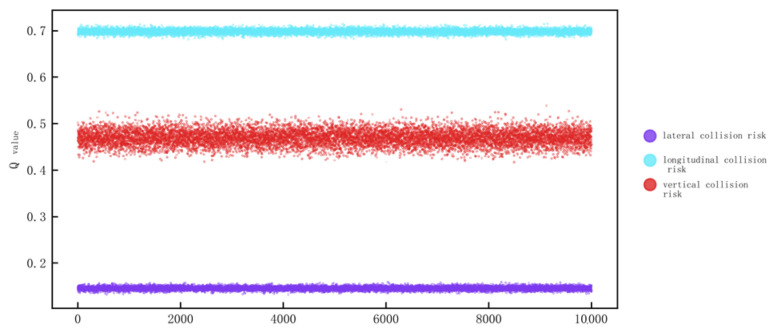
Q-values of the enhanced EMGF-M model in longitudinal, lateral, vertical directions.

The Q-values obtained using the QMC-S model were compared with those obtained using the traditional MC model. Taking rectangular prisms, spheres, and the MGFPE improved model as examples, the calculation results are shown in Figure 5:

As can be seen from the above figure, the use of the QMC-S model can reduce errors generated during Q-value calculations, resulting in more accurate computational results. The optimized Q-value improves the accuracy of collision risk calculations. Therefore, the established EMGF-M model is applied to multi-dimensional Q-value calculations, with the results shown in Figure 6. After averaging, the Q-values obtained for the EMGF-M improved model in the longitudinal, lateral, and vertical directions are: 0.469 667, 0.145 764, and 0.698 31, respectively.

To further demonstrate the feasibility of the improved EMGF-M model, its accuracy in calculating multi-dimensional collision risk values was compared with that of traditional rectangular prism collision models and spherical collision models.

**Figure 7:**
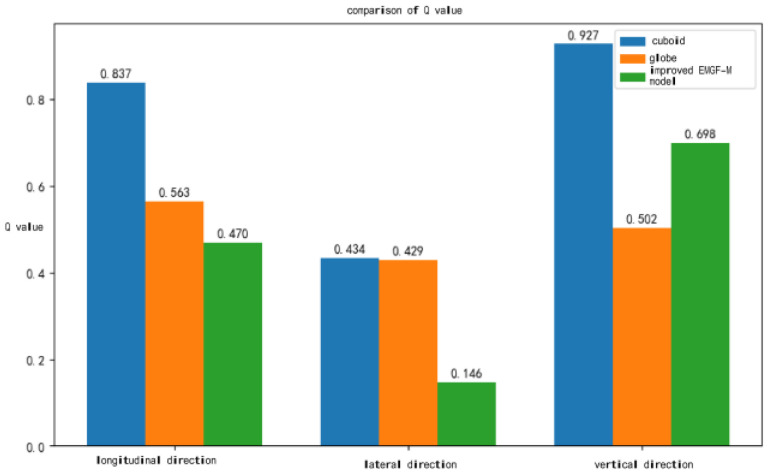
Comparison of Q values of three models in three directions.

After verification, the Q-values of the three models were compared in multiple dimensions, as shown in Figure 7. In terms of longitudinal collisions, the Q-values of the rectangular prism, sphere, and EMGF-M improved model were 0.837, 0.563, and 0.470, respectively. In lateral collisions, the Q-values for the rectangular prism, sphere, and EMGF-M improved model are 0.434, 0.429, and 0.146, respectively; In vertical collisions, the Q-values for the rectangular prism, sphere, and EMGF-M improved model are 0.927, 0.502, and 0.698, respectively.

As shown in Figure 7, the EMGF-M improved model exhibits higher Q-values in multiple dimensions compared to the cuboid and spherical models. Integrating the Q-values of all three models, the accuracy comparison results are presented in Table 2.

Based on the analysis of accuracy comparison values, it can be seen that the EMGF-M improved model outperforms the rectangular model in terms of Q-value calculation results in both the longitudinal and lateral directions, with an accuracy improvement of 45% compared to the rectangular model and 40.6% compared to the spherical model. In summary, the EMGF-M improved model demonstrates significant computational advantages and provides the necessary model performance foundation for safety margin calculations, as summarized in Table 3.

## 3. Improve Event Model Collision Risk Calculation

### 3.1. Improved Longitudinal Collision Risk Calculation

**Figure 8:**
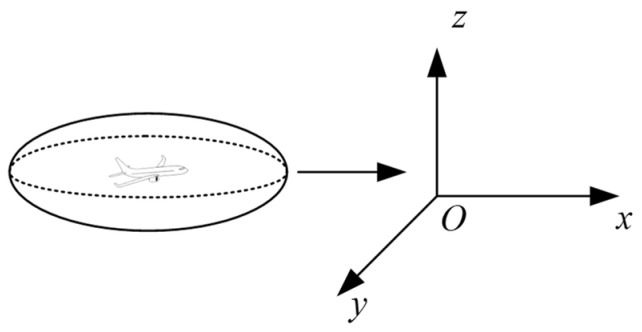
Improved longitudinal collision modeling.

**Figure 9:**
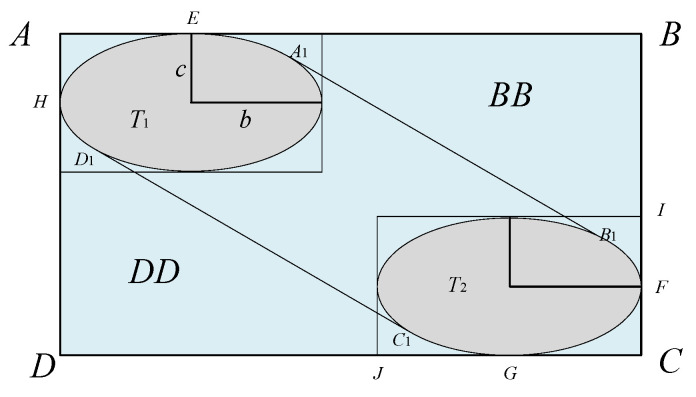
Improved Longitudinal Expansion Collision Box.

The improved Event longitudinal collision risk model is shown in Figure 8:

When the collision box enters the spacer layer area and passes through the spacer layer, the extended collision box generated is ABCD, as shown in Figure 9:

In Figure 9, ABCD represents the improved longitudinal extended collision box, A_1_B_1_C_1_D_1_ represents the area where the collision box passes through the spacer layer. The area of the extended collision box is S_ABCD_, the area where it passes through the spacer layer is S_A1B1C1D1_, and the time it takes for the collision box to pass through the spacer layer is *t*, $t=\frac{2a}{u_{x}}$, $DJ=u_{y}\times \frac{2a}{u_{x}}$, $BI=u_{z}\times \frac{2a}{u_{x}}$, JC=2b, IC=2c, The area of the collision box is expanded to

(3) $$S_{ABCD}=BC\times DC=(BI+IC)(DJ+JC)=(u_{z}\times \frac{2a}{u_{x}}+2b)(u_{y}\frac{2a}{u_{x}}+2c)$$

Center coordinates:

(4) $$\left(b,u_{z}\frac{2a}{u_{x}}+c),(u_{y}\frac{2a}{u_{x}}+b,c\right)$$

Elliptic equation:

First elliptic T1 equation:

(5) $$\frac{{\left(x-b\right)}^{2}}{b^{2}}+\frac{{\left(y-u_{z}\frac{2a}{u_{x}}-c\right)}^{2}}{c^{2}}=1$$

Second elliptic T2 equation:

(6) $$\frac{{\left(x-u_{y}\frac{2a}{u_{x}}-b\right)}^{2}}{b^{2}}+\frac{{\left(y-c\right)}^{2}}{c^{2}}=1$$

Slope of a straight line:

(7) $$k=-\frac{u_{z}}{u_{y}}$$

Equations of two tangents:

Equation of tangent A_1_B_1_:

(8) $$y_{1}=-\frac{u_{z}}{u_{y}}x+p_{1}$$

Equation of tangent C_1_D:

(9) $$y_{2}=-\frac{u_{z}}{u_{y}}x+p_{2}$$

Substitute the tangent equation into the first elliptical equation and convert it to standard form:

(10) $$A_{1}x^{2}+B_{1}x+C_{1}=0$$

(11) $$A_{1}=\frac{1}{b^{2}}+\frac{{u_{z}}^{2}}{{u_{y}}^{2}c^{2}}$$

(12) $$B_{1}=-\frac{2}{b}-\frac{2\frac{u_{z}}{u_{y}}(p_{1}-u_{z}\frac{2a}{u_{x}}-c)}{c^{2}}$$

(13) $$C_{1}=\frac{{\left(p_{1}-u_{z}\frac{2a}{u_{x}}-c\right)}^{2}}{c^{2}}$$

Substitute into the second elliptic equation and convert to standard form:

(14) $$A_{2}x^{2}+B_{2}x+C_{2}=0$$

(15) $$A_{2}=\frac{1}{b_{2}}+\frac{{u_{z}}^{2}}{{u_{y}}^{2}c^{2}}$$

(16) $$B_{2}=-\frac{2(u_{y}\frac{2a}{u_{x}}+b)}{b^{2}}-\frac{2\frac{u_{z}}{u_{y}}(p_{1}-c)}{c^{2}}$$

(17) $$C_{2}=\frac{{\left(u_{y}\frac{2a}{u_{x}}+b\right)}^{2}}{b^{2}}+\frac{{\left(p_{1}-c\right)}^{2}}{c^{2}}-1$$

Use the quadratic formula to solve for two solutions:

(18) $$x_{1,2}=\frac{-B\pm \sqrt{B^{2}-4AC}}{2A}$$

(19) $$y_{1,2}=-\frac{uz}{uy}x_{1,2}+p_{1,2}$$

Obtain the coordinates of the intersection point through calculation:

(20) $$\left(x_{1},y_{1})=(\frac{-B_{1}+\sqrt{{B_{1}}^{2}-4A_{1}C_{1}}}{2A1},-\frac{uz}{uy}\cdot \frac{-B_{1}+\sqrt{{B_{1}}^{2}-4A_{1}C_{1}}}{2A1}+p_{1}\right)$$

(21) $$\left(x_{2},y_{2})=(\frac{-B_{1}-\sqrt{B1^{2}-4A_{1}C_{1}}}{2A1},-\frac{uz}{uy}\cdot \frac{-B_{1}-\sqrt{{B_{1}}^{2}-4A_{1}C_{1}}}{2A_{1}}+p_{1}\right)$$

(22) $$\left(x_{3},y_{3})=(\frac{-B_{2}+\sqrt{{B_{2}}^{2}-4A_{2}C_{2}}}{2A_{2}},-\frac{uz}{uy}\cdot \frac{-B_{2}+\sqrt{{B_{2}}^{2}-4A_{2}C_{2}}}{2A_{2}}+p_{2}\right)$$

(23) $$\left(x_{4},y_{4})=(\frac{-B_{2}-\sqrt{{B_{2}}^{2}-4A_{2}C_{2}}}{2A_{2}},-\frac{uz}{uy}\cdot \frac{-B_{2}-\sqrt{{B_{2}}^{2}-4A_{2}C_{2}}}{2A_{2}}+p_{2}\right)$$

Calculate the ratio of the area enclosed by two ellipses and two tangents to the total area using the QMC-S method, and calculate the probability Q that aircraft B is located within the longitudinal extended collision box.

Longitudinal collision risk calculation process:

(24) $$N_{x}=2f_{{GERH}_{x}}\times p_{y}\times p_{z}$$

(25) $$f_{{GERH}_{x}}=\frac{p_{x}(S_{x})}{t_{x}}=p_{x}(S_{x})\frac{u_{x}}{2a}$$

(26) $$p_{y}=E(0)\times \frac{b}{S_{y}}(1+\frac{2au_{y}}{2bu_{x}})$$

(27) $$p_{z}=p_{z}(0)(1+\frac{2au_{z}}{2cu_{x}})$$

Final expression for longitudinal collision risk:

(28) $$Nx^{\prime }=2p_{x}(S_{x})\times E(0)\times p_{z}(0)\times \frac{b}{S_{y}}\times \frac{u_{x}}{2a}\times (1+\frac{2au_{y}}{2bu_{x}})\times (1+\frac{2au_{z}}{2cu_{x}})\times Q$$

A simplified derivation of the longitudinal collision risk model, including the geo-metric basis, the QMC-S method, and the final expression, is provided in Appendix A.

### 3.2. Improved Side Impact Risk Model

**Figure 10:**
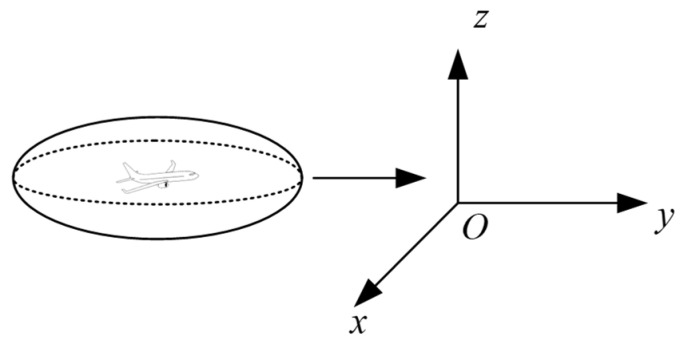
Improved lateral collision risk modeling.

**Figure 11:**
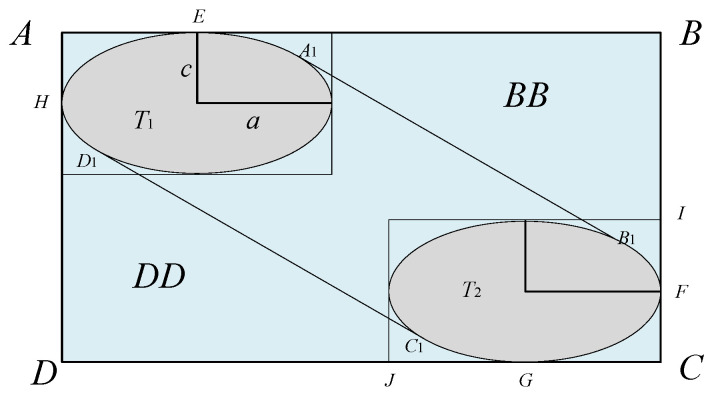
Improved lateral Expansion Collision Box.

The improved Event lateral collision risk model is shown in Figure 10. When the collision box enters the interval layer area and crosses the interval layer, the extended collision box generated is ABCD, as shown in Figure 11:

Center coordinates:

(29) $$\left(a,u_{z}\frac{2b}{u_{y}}+c),(u_{x}\frac{2b}{u_{y}}+a,c\right)$$

Elliptic equations:

First elliptic T1 equation:

(30) $$\frac{{\left(x-a\right)}^{2}}{a^{2}}+\frac{{\left(y-u_{z}\frac{2b}{u_{y}}-c\right)}^{2}}{c^{2}}=1$$

Second elliptic T2 equation:

(31) $$\frac{{\left(x^{2}-u_{x}\frac{2b}{u_{y}}-a\right)}^{2}}{a^{2}}+\frac{{\left(y-c\right)}^{2}}{c^{2}}=1$$

Slope:

(32) $$k=-\frac{u_{z}}{u_{x}}$$

Tangent equation:

Equation of tangent A_1_B_1_:

(33) $$y_{3}=-\frac{u_{z}}{u_{x}}x+p_{1}$$

Equation of tangent C_1_D_1_:

(34) $$y_{4}=-\frac{u_{z}}{u_{x}}x+p_{2}$$

From Section 2.3, the final expression for the improved lateral collision risk is obtained as:

(35) $${N_{y}}^{\prime }=2p_{y}(S_{y})\times E(0)\times p_{x}(0)\times \frac{c}{S_{z}}\times \frac{u_{y}}{2b}\times (1+\frac{2bu_{z}}{2cu_{y}})\times (1+\frac{2bu_{z}}{2au_{y}})\times Q$$

### 3.3. Improving the Vertical Collision Risk Model

**Figure 12:**
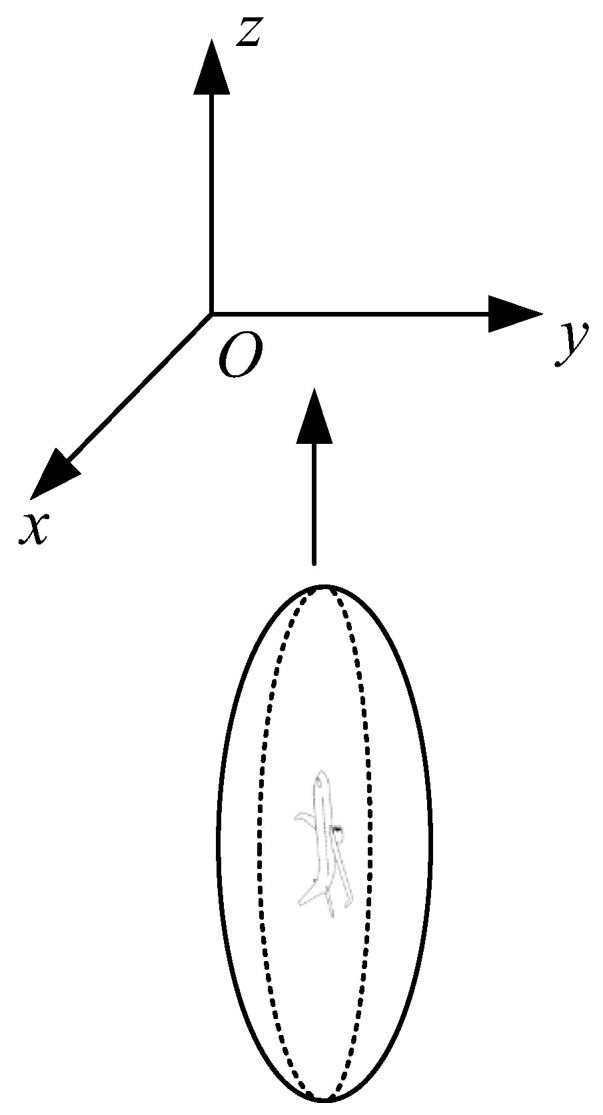
Improved vertical collision risk modeling.

**Figure 13:**
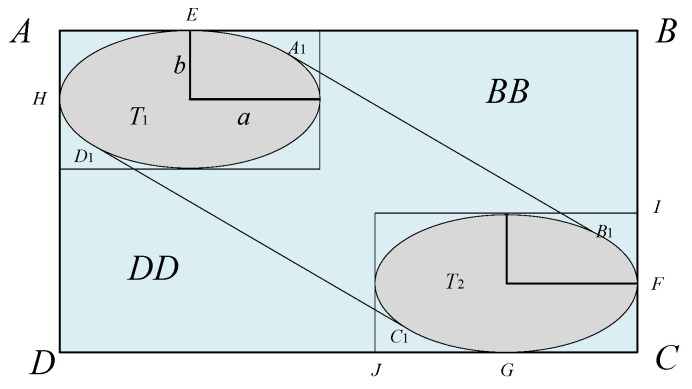
Improved vertical collision risk modeling.

The improved Event vertical collision risk model is shown in Figure 12. When the collision box enters the interval layer area and crosses the interval layer, the extended collision box generated is ABCD, as shown in Figure 13:

Vertical direction:

(36) $$\left(a,u_{y}\frac{2c}{u_{z}}+b),(u_{x}\frac{2c}{u_{z}}+a,b\right)$$

Ellipse equation:

First ellipse T1 equation:

(37) $$\frac{{\left(x-a\right)}^{2}}{a^{2}}+\frac{{\left(y-u_{y}\frac{2c}{u_{z}}-b\right)}^{2}}{b^{2}}=1$$

Second ellipse T2 equation:

(38) $$\frac{{\left(x-u_{x}\frac{2c}{u_{z}}-a\right)}^{2}}{a^{2}}+\frac{{\left(y-b\right)}^{2}}{b^{2}}=1$$

Slope:

(39) $$k=-\frac{u_{x}}{u_{y}}$$

Tangent equation:

Tangent A_1_B_1_ equation:

(40) $$y_{5}=-\frac{u_{x}}{u_{y}}x+p_{1}$$

Tangent C_1_D equation:

(41) $$y_{6}=-\frac{u_{x}}{u_{y}}x+p_{2}$$

As can be seen from Section 2.3, the final expression for the vertical collision risk is as follows:

(42) $${N_{z}}^{\prime }=2p_{z}(S_{z})\times E(0)\times p_{y}(0)\times \frac{a}{S_{x}}\times \frac{u_{z}}{2c}\times (1+\frac{2cu_{x}}{2au_{z}})\times (1+\frac{2cu_{y}}{2bu_{z}})\times Q$$

## 4. Experimental Simulation and Analysis

### 4.1. Parameter Settings

The operational conditions of small and medium-sized airports in the southwest region and the airspace structure were selected as the simulation experiment scenario. The manned aircraft selected was the Airbus A320 model, and the unmanned aircraft selected was the Double Scorpion model. The flight performance, rated speed, and collision box dimensions, as well as the overlap probability and speed in the longitudinal, lateral, and vertical directions, are shown in Table 4 and Table 5.

### 4.2. Safety Clearance Calculation

**Figure 14:**
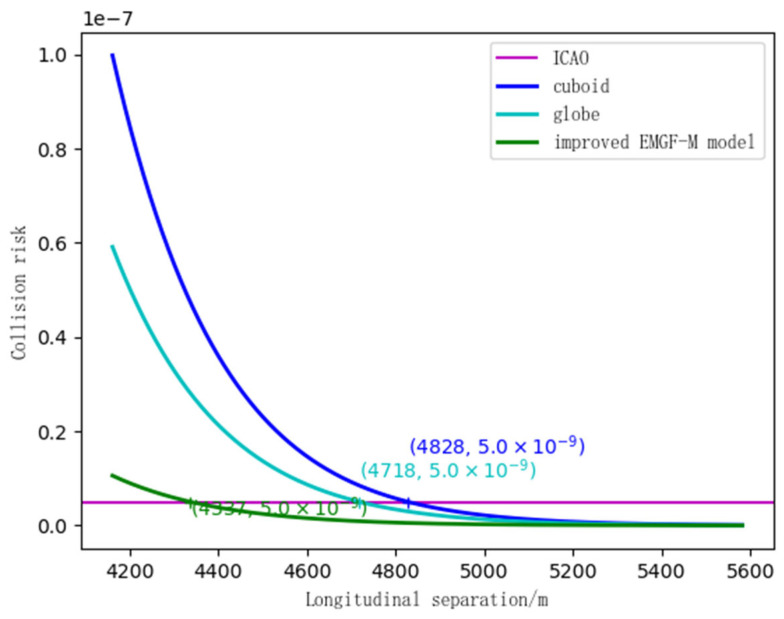
Three model longitudinal safety intervals.

**Figure 15:**
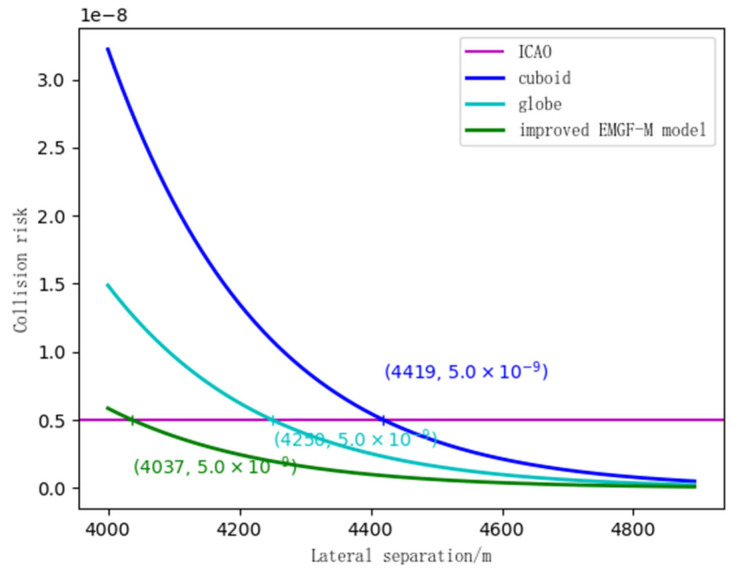
Three model lateral safety intervals.

**Figure 16:**
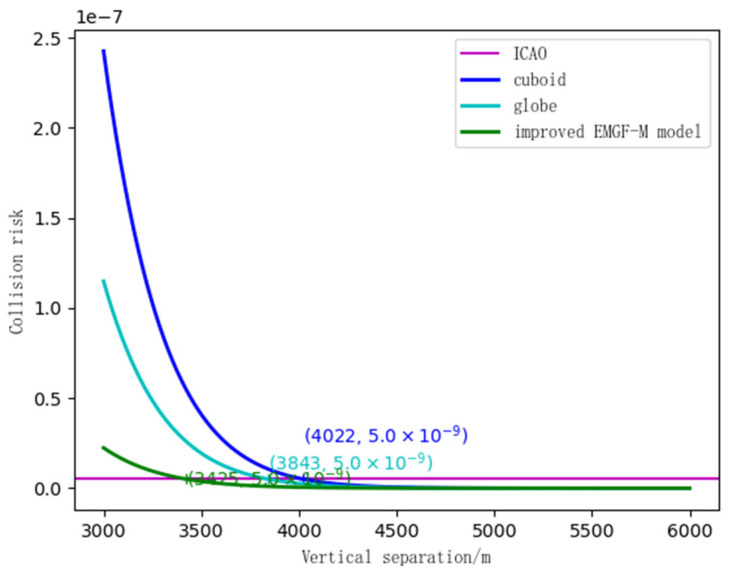
Three model vertical safety intervals.

Using simulated experimental scene information, a collision risk calculation was conducted for the improved EMGF-M model and traditional models. Combined with the safety target levels proposed by the International Civil Aviation Organization (ICAO), the calculations were performed respectively based on Equations (27), (34), and (41). This process yielded the comparison ratios between the safety intervals in the longitudinal, lateral, and vertical directions—considering multi-dimensional spatial geometric features and flight performance—and the results from other models. The findings are presented in Figure 14, Figure 15 and Figure 16.

In Figure 14, the horizontal line represents the safety target level, while the curve indicates the longitudinal collision risk corresponding to each model. From the figure, it can be observed that the initial collision risk value for the cuboid is relatively high, approximately 1.0 $\times \;10^{-7}$. When the separation distance reaches 4828 m, the collision risk decreases to the safety target level. The initial collision risk for the sphere is about 0.6 × $10^{-7}$, which is slightly lower than that of the cuboid. As the longitudinal separation increases, the collision risk continues to decline, reaching the safety target level at a separation of 4718 m. The improved EMGF-M model exhibits the lowest initial collision risk among the three, approximately 0.1 × $10^{-7}$, and reaches the safety target level at a separation of 4329 m.

In Figure 15, the horizontal line is the safety target level, and the curves are the lateral collision risks corresponding to each model. It can be seen from Figure 15 that the initial collision risk of the cuboid is 3.0 × $10^{-8}$. When the interval reaches 4419 m, the collision risk drops to the safety target level. The initial collision risk of the sphere is about 1.5 × $10^{-8}$. As the lateral interval increases, the collision risk gradually decreases. When the interval is 4250 m, the collision risk is at the safety target level. The initial collision risk of the EMGF-M improved model is 0.6 × $10^{-8}$, and it reaches the safety target level at 4026 m.

In Figure 16, the horizontal line shows the safety target level, and the curves show the collision risk in the vertical direction for different models. For the rectangular box, the initial collision risk is 2.5 × $10^{-7}$. When the distance reaches 4022 m, the collision risk drops to a safe level. For the sphere, the initial risk is about 1.2 × $10^{-7}$, and at 3843 m, it reaches the safe level. The improved EMGF-M model starts with a risk of 0.4 × $10^{-7}$, and at 3450 m, it becomes safe.

Based on the above, at the initial stage in all three directions, the collision risk value for the rectangular box is higher, while the EMGF-M improved model has a lower risk. To meet the safety target level, the calculated safe interval values differ in terms of distance and sensitivity. Compared to the rectangular box and sphere, the EMGF-M model can further reduce the interval values under the same safety standard, which can lead to better airspace utilization. The results are shown in Table 6.

From the comparison in Table 6, it can be seen that the safety interval values provided by the EMGF-M improved model are better than those for the rectangular box and sphere. It performs better in aspects such as mathematical accuracy and model stability. Therefore, a multi-dimensional spatial safety interval of 4329 m that meets the safety target level has been established, and it is rounded up to 4500 m to facilitate better integration and planning of flight trajectories in the next step.

### 4.3. Integrated Flight Program Design Application

The operational conditions and airspace structure of small and medium-sized airports in the Southwest Region were selected as the experimental scenario. The EMGF-M enhanced model was utilized to apply optimized separation minima for hybrid airspace planning, establishing and validating integrated flight procedures. By implementing a departure and arrival segregated operation mode for transition flights between two general aviation airports, a UAV-manned aircraft integrated flight trajectory design was achieved under the current effective airspace structure. The hybrid airspace integration operational scheme was further optimized and verified based on separation minima.

**Figure 17:**
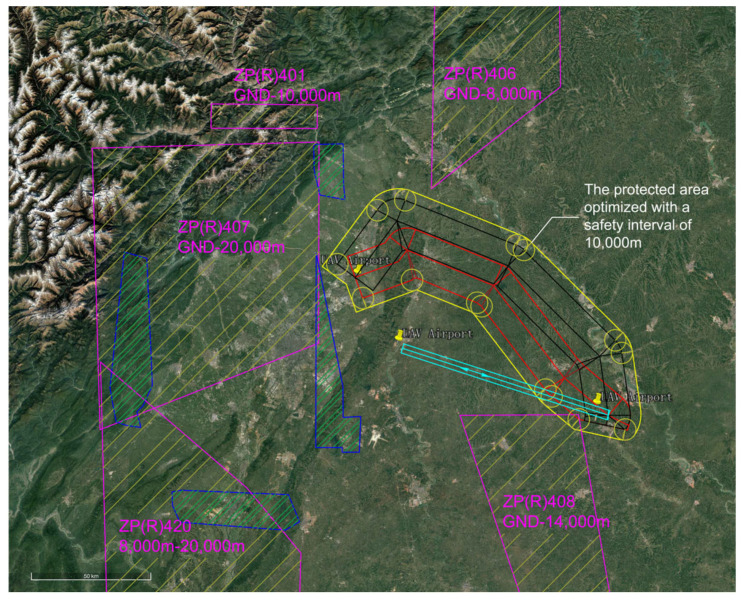
Primary airspace and protected area boundaries.

The restricted areas and civil aviation flight procedure protection zones within the current effective airspace were delineated in the experimental scenario. Due to constraints from airspace restriction zoning and civil aviation flight procedure design, only a unidirectional UAV route was provisioned between the two airports. The volume of routine flight trajectories and traffic flow was significantly constrained. The current effective airspace structure and unidirectional UAV route are illustrated in Figure 17.

**Figure 18:**
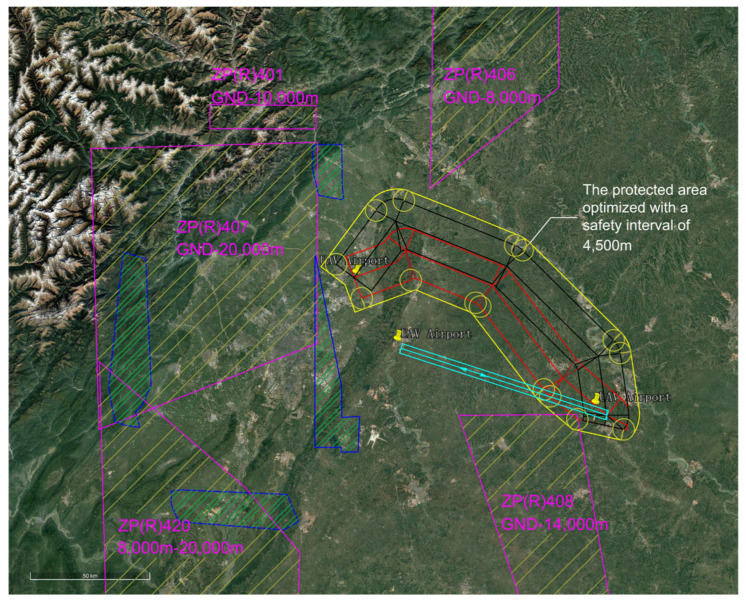
Optimized airspace and protected area boundaries.

**Figure 19:**
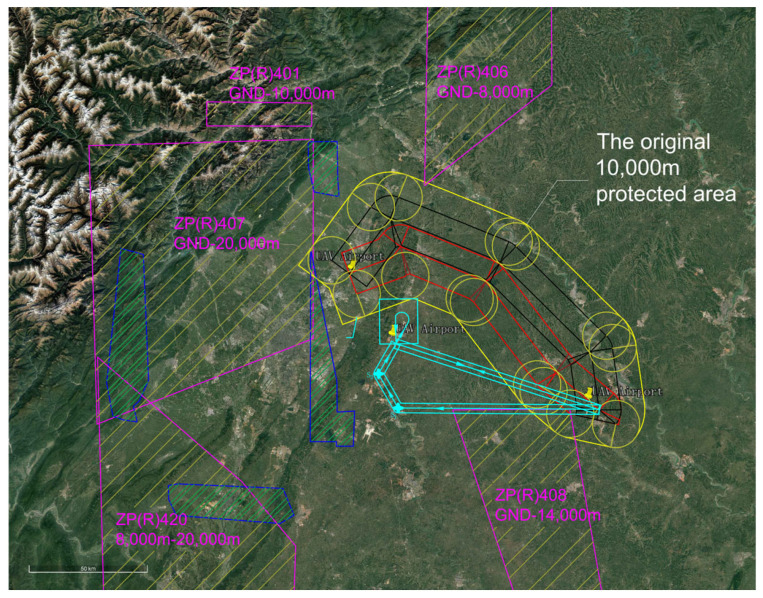
Pre-optimization Separation Approach and Departure Modes.

**Figure 20:**
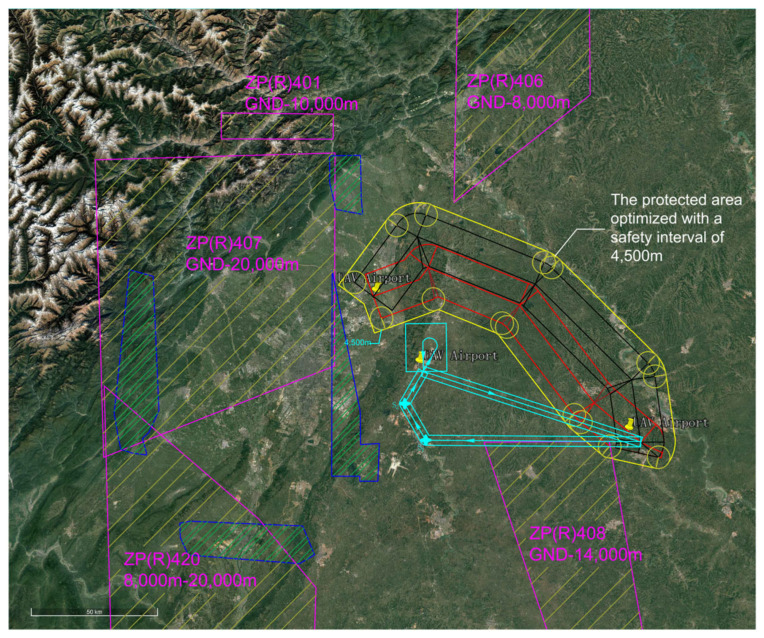
Optimized Separate Approach and Departure Modes.

**Figure 21:**
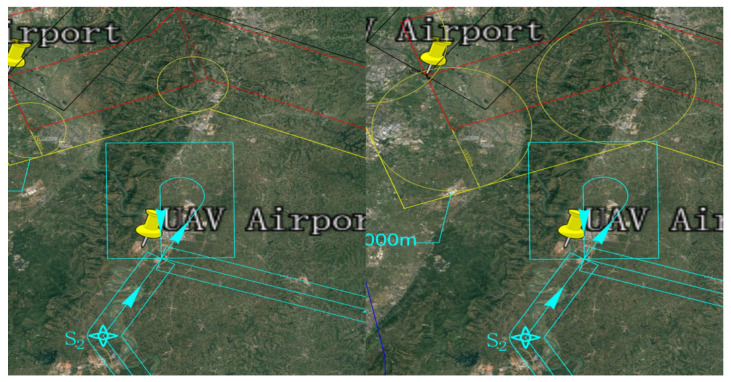
Programming Optimization Before and After Comparison.

The EMGF-M enhanced model calculated the integrated flight safety risk compliant with ICAO safety target levels and trajectory planning separation minima. An optimized design was implemented for the airspace depicted in Figure 17. Based on the derived separation minima, the protection zone boundaries and redundancy within the current effective airspace were redefined, as shown in Figure 18. A hybrid airspace integration flight procedure scheme was proposed for the two airports according to the new protection zones. A base turn procedure compliant with separation minima was introduced to enable segregated departure and arrival operations between the airports. To demonstrate the reduction in airspace redundancy resulting from separation minima, protection zone diagrams for the base turn procedure without and with separation standards were plotted (Figure 19 and Figure 20). Figure 20 shows no overlap between the base turn protection zone and existing restricted areas, unlike Figure 19, confirming feasibility for segregated operations (see Figure 21 for a detailed view).

Taking the hybrid airspace integration flight procedure design for Southwest regional airports as an example, the pre-optimization route length between airports was 92,600 m. Post-implementation of segregated departure and arrival procedures, it extended to 209,000 m. Per the “Interim Regulations on Unmanned Aircraft Flight Management”, UAVs must maintain ≥10 km (5.4 NM) separation from manned aircraft or comply with ATC instructions. Applying a dynamic separation-based capacity assessment method [16], traffic flow on the new trajectory was calculated. Post-implementation traffic flow increased from 9.2 flights to 20.9 flights.

The comparative results of airspace procedure layouts before and after optimization based on safety intervals derived from the EMGF-M model are illustrated in Figure 21. Prior to optimization (left panel), significant overlap exists between the baseline turn procedure protection zone and the existing airway protection zone, indicating that the original separation criteria could not ensure safe segregated operations. After applying the optimized safety intervals calculated by the model (right panel), the two zones are fully separated with no spatial conflict, demonstrating that the refined separation standards effectively support segregated operations and enhance both airspace utilization efficiency and operational safety (see Figure 21 for details).

## 5. Conclusions

(1) This paper introduces a QMC-S model to establish an enhanced EMGF-M model that addresses geometric characteristics in the spatial domain and flight performance. The proposed model improves computational stability and demonstrates a significant increase in accuracy compared to conventional models.
(2) The integration of the improved EMGF-M model with ICAO Safety Level objectives enables a comprehensive risk analysis in mixed airspace, yielding refined and quantifiable spatial safety separations.
(3) A simulation of mixed airspace procedure design and traffic flow calculation was conducted for regional airports in Southwest China, utilizing the safety intervals derived from the improved EMGF-M model, which resulted in a 2.18-fold enhancement in traffic flow.

Despite the contributions above, this study has several limitations. First, the proposed model is validated using only a single scenario (small- and medium-sized airports in Southwest China), which may limit the generalizability of the findings to other airspace structures or traffic patterns. Second, the simulation considers only traffic flow as the evaluation index; other critical factors such as communication latency, wind field disturbances, and emergency conditions are not incorporated. Third, the ellipsoid-based collision box, while more accurate than cuboid and spherical models, still relies on simplified geometric assumptions that may not fully capture complex UAV aerodynamic behaviors.

Future research can be extended in several directions. First, the model should be validated using multiple scenarios with varying airspace structures, traffic densities, and environmental conditions to enhance its robustness and generalizability. Second, additional evaluation metrics—such as communication integrity, collision avoidance success rate, and operational cost—can be introduced to provide a more comprehensive safety assessment. Third, the current model can be extended to consider emergency situations (e.g., engine failure, loss of communication) and dynamic environmental factors (e.g., wind gusts, visibility constraints). Furthermore, with the rapid development of urban air mobility, the applicability of the proposed model to eVTOL and autonomous swarm operations warrants further investigation.

## Figures and Tables

**Table 1:** Summary of the characteristics of current collision risk research methods.

Research Category	Representative Literature	Core Research Characteristics
Improvements Based on the Reich Model	[3,4,5,6,7,8,9,10,11]	Introduces new parameters (e.g., vertical risk, position error probability, random velocity distribution, CNS performance) into classical collision models, enhancing model sensitivity under specific factors.
Comprehensive Risk Assessment Models	[12,13]	Considers multiple factors, including UAV performance, airspace conditions, operational modes, and human factors, to construct a multi-factor coupled risk assessment framework.
Safety Separation Studies for Specific Aircraft Types	[14,15,16]	Targets specific UAV types (e.g., logistics UAVs, multi-rotor UAVs, eVTOL) and specific scenarios (e.g., urban low-altitude airspace) to identify key risk factors and calculate safety separations.

**Table 2:** Comparison of error results.

Category	Mean Error	Standard Deviation of Error
MC	$3.89\times 10^{-3}$	$2.51\times 10^{-3}$
QMC-S	$8.11\times 10^{-5}$	$6.09\times 10^{-5}$
QMC-S	$8.11\times 10^{-5}$	$6.09\times 10^{-5}$

**Table 3:** MGFPE Improved Model Accuracy Contrasts.

Collision Direction	Percentage Advantage of Relative Cuboids/%	Relative Ball Advantage Percentage/%
Longitudinal collision	44.91%	16.64%
Lateral collision	64.40%	66.03%
Vertical collision	27.67%	39.08%
Comprehensive accuracy improvement	45.00%	40.60%

**Table 4:** Flight Performance Parameters.

Category	AirbusA320	Double-Tailed Scorpion Drone
Wingspan/m	34.1	20
Fuselage length/m	37.75	10
Fuselage height/m	11.76	3.1
Maximum takeoff weight/ton	73.5	2.8
Maximum payload/ton	16.3	1–1.2
Maximum range/km	5000	6
Maximum speed/(km·h^−1^)	925	300
Normal cruising speed/(km·h^−1^)	828	200–250
Service ceiling/m	12,000	6000
Takeoff roll distance/m	2090	700

**Table 5:** Calculation parameters.

Parameter	Parameter Value	Parameter	Parameter Value
u*_x_*/(m·s^−1^)	230	2a/m	37.75
u*_y_*/(m·s^−1^)	6.5	2b/m	34.1
u*_z_*/(m·s^−1^)	0.8	2c/m	11.76
E(0)	0.01		

**Table 6:** The result of the calculation of the safety interval value.

Collision Direction	Safety Interval Value		
	Cuboid	Sphere	EMGF-M
Longitudinal	4828	4718	4329
Lateral	4419	4250	4026
Vertical Direction	4022	3843	3450

## Data Availability

Dataset available upon request from the authors.

## Appendix A. Derivation of Longitudinal Collision Risk

This appendix provides a simplified derivation of the longitudinal collision risk model EMGF-M presented in Section 2.1 of the main text. The focus is on the core logic and key results, with detailed algebraic expansions omitted as per the reviewer’s suggestion for conciseness.

### Appendix A.1. Geometric Basis

In the EMGF-M model, Aircraft A is represented as an ellipsoidal collision box with semi-axes a (length), b (width), and c (height). Aircraft B is treated as a point mass. When Aircraft A traverses the separation layer with relative velocities $u_{x},u_{y}\;and\;u_{z}$ in the longitudinal, lateral, and vertical directions respectively, an extended collision box is generated.

The time required for the collision box to completely cross the separation layer is

$$t=\frac{2a}{u_{x}}$$

During this interval, lateral and vertical displacements occur due to relative motion:

$$\Delta y=u_{y}\cdot \frac{2a}{u_{x}},\;\Delta z=u_{z}\cdot \frac{2a}{u_{x}}$$

The total area of the extended collision box ABCD is therefore

$$S_{ABCD}=\left(\Delta y+2b\right)\left(\Delta z+2c\right)=\left(u_{y}\cdot \frac{2a}{u_{x}}+2b\right)\left(u_{z}\cdot \frac{2a}{u_{x}}+2c\right)$$

The region of interest for collision risk is the intersection area $S_{cross}$—the area enclosed by two ellipses and their common tangents within the extended collision box. This geometry is illustrated in Figure 9 of the main text.

### Appendix A.2. Calculation of Probability Q

The probability Q is defined as the likelihood that Aircraft B lies within the extended collision box. Geometrically, this equals the ratio of the intersection area $S_{cross}$ to the total extended box area $S_{ABCD}$:

$$Q=\frac{S_{cross}}{S_{ABCD}}$$

Direct analytical solution of $S_{cross}$ involves solving multiple elliptic equations and tangent intersections—a mathematically complex process with limited additional insight. The EMGF-M model therefore employs the QMC-S method (Quasi-Monte Carlo with scrambling) described in Section 2.3 of the main text to compute this area ratio numerically.

Table A1 compares the computational performance of the QMC-S method against traditional Monte Carlo (MC) simulation.

**Table A1:** Error comparison: MC vs. QMC-S methods.

Method	Mean Error	Standard Deviation of Error
MC	3.89 × 10^−3^	2.51 × 10^−3^
QMC-S	8.11 × 10^−5^	6.09 × 10^−5^

As shown in Table A1, the QMC-S method reduces the mean error by 97.91% and the standard deviation by 97.57% compared to traditional MC simulation, demonstrating its suitability for accurate Q-value calculation.

Applying the QMC-S method to the EMGF-M model yields the following Q-values for the three dimensions:

These values serve as refined probability inputs for the collision risk calculations in Section 3.2.

### Appendix A.3. Derivation Summary and Final Expression

Following the standard Event model framework, the longitudinal collision risk $N_{x}$ is constructed through the following steps:

1. Interval loss frequency: The frequency with which aircraft lose longitudinal separation is $2p_{x}\cdot \left(S_{x}\right)\cdot \frac{u_{x}}{2a},$ where $p_{x}\left(S_{x}\right)$ represents the proportion of aircraft pairs at longitudinal spacing $S_{x}$.
2. Overlap probabilities: The probability that aircraft are vertically aligned is $E\left(0\right)$. Given vertical alignment, the lateral overlap probability is $P_{z}\left(0\right)$ adjusted by the geometric factor $\frac{b}{S_{y}}$.
3. Motion compensation factors: During the interval crossing time, relative motion in the lateral and vertical directions contributes additional exposure, captured by the factors $\left(1+\frac{2au_{y}}{2bu_{x}}\right)$ and $\left(1+\frac{2au_{z}}{2cu_{x}}\right)$.
4. Q-value integration: The probability that Aircraft B lies within the extended collision box, Q, multiplies all preceding factors to yield the final collision risk.

Combining these elements gives the complete expression for longitudinal collision risk:

$$N_{x}=2p_{x}\left(S_{x}\right)\times E\left(0\right)\times p_{z}\left(0\right)\times \frac{b}{S_{y}}\times \frac{u_{x}}{2a}\times \left(1+\frac{2au_{y}}{2bu_{x}}\right)\times \left(1+\frac{2au_{z}}{2cu_{x}}\right)\times Q$$

All parameters are defined in Section 2.1 of the main text. The key enhancement of the EMGF-M model lies in:

- The ellipsoidal collision geometry (reflected in the motion compensation factors)
- The QMC-S-based calculation of Q (detailed in Appendix A.2)
- The resulting refinement of probability inputs (Table A2)

**Table A2:** Q-values for each dimension.

Direction	Q-Value
Longitudinal $\left(Q_{x}\right)$	0.469667
Lateral $\left(Q_{y}\right)$	0.145764
Vertical $\left(Q_{z}\right)$	0.698310

## References

[1] Lu F., Yan K., Li W., Wang F. (2024). Overall architecture and enhancement pathways of low-altitude economy. Inf. Commun. Technol. Policy.

[2] Zhang K. (2024). Functions of Government Agencies in Low-Altitude Management. J. Beijing Univ. Aeronaut. Astronaut. Soc. Sci. Ed..

[3] Reich P.G. (1966). Analysis of long-range air traffic systems: Separation standards—I. J. Navig..

[4] Brooker P. (2003). Lateral collision risk in air traffic track systems: A ‘post-Reich’ event model. J. Navig..

[5] Bai H., Hsu D., Kochenderfer M.J., Lee W.S. (2012). Unmanned aircraft collision avoidance using continuous-state POMDPs. Robotics: Science and Systems.

[6] Zhang Z., Zhang J., Wang P., Chen L. (2018). Research on Operation of UAVs in Non-isolated Airspace. Comput. Mater. Contin..

[7] Deng L. (2019). Research of collision probability of unmanned aerial vehicles and civil airplane. J. Nanjing Univ. Sci. Technol..

[8] Wang L., Yang J. (2022). Research on assessment method of safety separation for logistics UAVs based on position error probability model. J. Saf. Sci. Technol..

[9] Wang L., Yang J. (2022). A Collision Risk Model for Small UAVs Based on Velocity Random Distribution in Low-altitude Airspace. J. Transp. Inf. Saf..

[10] Gao Y., Liu D. (2014). Research on Aircraft Collision Risk Model in Terminal Area after Low-altitude Opening. China Saf. Sci. J..

[11] Gao J. (2018). Research on Safety Flight Risk Assessment of Unmanned Aerial Vehicles. Master’s Thesis.

[12] Li N., Sun L., Jiao Q., Zheng Z., Liu Y. (2024). The Method for Determining the Safety Separation of Unmanned Aerial Vehicles. Sci. Technol. Eng..

[13] Zhang K., Huang L., He Y., Wang B., Chen J., Tian Y., Zhao X. (2023). A real-time multi-ship collision avoidance decision-making system for autonomous ships considering ship motion uncertainty. Ocean. Eng..

[14] Li H., Nie F. (2023). Collision Risk Assessment of Logistics UAV Based on Bayesian Network. Sci. Technol. Eng..

[15] Zhang H., Li B., Liu H., Zhong G., Fei Y. (2023). Demarcation method of safety separation for multi-rotor UAV in free airspace. Syst. Eng. Electron..

[16] Wang X., Wang Y. (2025). Safety interval evaluation for multi-aircraft eVTOL inurban low altitude. Acta Aeronaut. Et Astronaut. Sin..

[17] Wang X., Cao W., Li T., Feng Y., Uğurlu Ö., Wang J. (2025). An integrated multidimensional model for heterogeneity analysis of maritime accidents during different watchkeeping periods. Ocean. Coast. Manag..

[18] Zhou Y., He R. (2024). Improvement of the Regulatory Legislation for Civil Unmanned Aerial Vehicles in China. J. Beijing Univ. Aeronaut. Astronaut. Soc. Sci. Ed..

[19] Chen X., Wu P., Wu Y., Aboud L., Postolache O., Wang Z. (2025). Ship trajectory prediction via a transformer-based model by considering spatial-temporal dependency. Intell. Robot..

